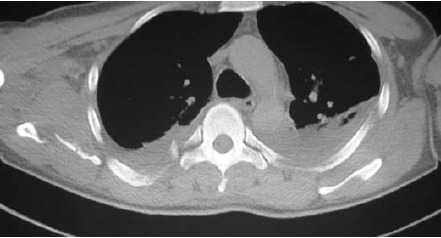# Scapulothoracic Dissociation: A Rare Variant: A Case Report

**DOI:** 10.5704/MOJ.1407.003

**Published:** 2014-07

**Authors:** Rajat Jangir, Diwakar Misra

**Affiliations:** Department of Orthopaedics, Rnt Medical College, Udaipur, Rajasthan, India; Department of Orthopaedics, Maulana Ajad Medical College, New Delhi, India

## Abstract

Scapulothoracic dissociation is a rare injury involving separation of scapula from the thorax along with the upper extremity. Majority of the patients have concomitant neurovascular injury and the prognosis is uniformly poor in such cases. We present a case of scapulothoracic dissociation with comminuted fracture of scapula and acromioclavicular joint disruption without neurovascular deficit. There were associated avulsion fractures of the spinous processes of vertebrae (T3-T5). Such presentation is rare in an already rare scapulothoracic dissociation injury. A discussion regarding the probable mechanism of injury, management and prognosis is presented.

**Key Words:**

scapulothoracic dissociation, scapula fracture, brachial plexus injury, vascular injury

## Introduction

Scapulothoracic articulation is formed by the muscles between the scapula and thorax with further linkage provided by the acromioclavicular joint- claviclesternoclavicular joint axis. Injuries to this articulation are rare ^1,2^ . Scapulothoracic dissociation is a devastating injury usually associated with neurovascular injury and a poor prognosis ^3^. We report a rare variety of scapulothoracic dissociation with comminuted scapular fracture and no neurological deficit.

## CASE REPORT

A 35 year old male patient presented to the emergency department with history of a road traffic accident with complaints of injury to chest and right shoulder. The patient was riding a bicycle when he was hit by a truck from behind. On examination, the patient was hemodynamically unstable.

There was massive swelling over the right shoulder with abrasions over the scapular region. Distal pulses were palpable. There was no neurological deficit. The patient was resuscitated with the ATLS (Advanced Trauma life Support) protocol.

Chest radiograph revealed multiple rib fractures and hemothorax. Right shoulder radiograph revealed acromioclavicular disruption, fracture of scapula and lateral translation of scapula. Intercostal drainage tubes were inserted for hemothorax. The right upper limb was strapped to the body. The electrocardiograph was suggestive of myocardial ischemia for which echocardiography was done. It revealed ejection fraction to be 30%. The patient was kept in the intensive care ward for 29 days for management of hemothorax and myocardial ischemia following which he was shifted to the orthopaedic wards and managed conservatively.

MRI imaging revealed altered signal intensity involving the rotator cuff -, deltoid, latissimus dorsi and pectoralis major muscles. Rotator cuff muscles showed hyper intensities suggesting partial tears. Computerized tomography revealed fracture of multiple spinous processes (T3-T5) with the fractured fragment drawn towards the side of the scapulothoracic dissociation demonstrating the avulsion fracture and direction of force. The patient was lost to follow-up.

## Discussion

The functional scapulothoracic joint is part of true closed chain with the acromioclavicular and the sternoclavicular joints. The term ‘scapulothoracic dissociation’ was coined by Oreck et al in 1984 to describe an injury involving complete closed separation of scapula and upper extremity from the thoracic attachments ^4^.

The mechanism of injury is usually a strong traction force applied to the shoulder girdle ^1, 4^. The traction force disrupts the muscular tissues and the acromioclavicular ligaments/ sternoclavicular ligaments making the neurovascular tissues vulnerable to injury. Deltoid, trapezius, levator scapulae, rhomboids, lattisimus dorsi and the pectoralis minor are partially or completely torn. The muscles mentioned give way before ligaments and vessels are damaged and before nerves. The strong traction force mechanism alone does not always explain the spectrum of injuries seen with scapulothoracic dissociation. Our patient had disruption of the acromioclavicular joint with comminuted fracture of scapula. The most probable mechanism of injury in our case was direct impact on the scapula which caused the fracture of scapula and then carried the scapula laterally resulting into scapulothoracic dissociation. Massive soft tissue swelling around the shoulder is classically present without breach in the skin. Usually the patients have multiple injuries and attention can be diverted easily to the more severe injuries like chest injury, head injury and other extremity fractures.

The diagnosis of scapulothoracic dissociation should be considered in a patient with the high energy trauma to upper limb with neurovascular deficit, radiograph demonstrating lateral displacement of the scapula or complete acromioclavicular disruption. The lateral displacement of scapula is measured in terms of distance between the spine and the medial border of scapula ^2^. Kelbel described the ratio of distances between affected and the non- affected sides to be 1.5 or greater.

Scapulothoracic dissociation is usually associated with other life threatening injuries. General principles of polytrauma care with cardiopulmonary stabilization and resuscitation should be of paramount importance. Life threatening injuries should be managed first and after the patient is stable, the shoulder is thoroughly investigated and a decision regarding the final treatment made. Further management is determined by any associated injuries and the patient’s neurovascular and haemodynamic status1. In haemodynamically stable patients, angiography is widely recommended prior to surgery. In haemodynamically unstable cases, however, urgent surgical intervention through high lateral thoracotomy, or median sternotomy, is required to control the arterial bleeding, as part of the resuscitation algorithm ^1^. This urgent surgery may include tamponade packing and emergency suturing to prevent exsanguinations.

Sampson et al. suggested a conservative policy towards revascularization for the arterial injury in scapulothoracic dissociation in view of infrequent occurrence of delayed hemorrhage and life threatening ischemia and dismal functional outcome of the brachial plexus injury ^3^. In those patients who require vascular repair, or other surgical interventions in the shoulder region, the brachial plexus should be explored in order to determine the degree of neurological injury. Lastly, orthopaedic stabilisation procedures in scapulothoracic dissociation are still controversial; however, the decision is based not only on the osseous and ligamentous injury patterns, but also on the concomitant neurovascular injuries, with respect to neurovascular repair, or reconstruction procedures ^1^.

Most studies involving patients with neurovascular damage have reported poor prognosis for these injuries ^5^. Zelle et al 20 regard the presence of a complete brachial plexus avulsion as predictive of a poor functional outcome in a patient with a scapulothoracic dissociation ^5^. If upper extremity function is not restorable, an early aboveelbow amputation and immediate prosthetic fitting should be performed, since this treatment approach results in superior functional outcomes ^1, 2^.

Figure 1

Figure 2

Figure 3

**Fig. 1: Radiograph showing lateral displacement of the scapula which can be quantified by the scapular index. The distance from the spinous process to the medial border of the scapula is measured bilaterally. The value of the injured side is divided by the value of the non-injured side.**
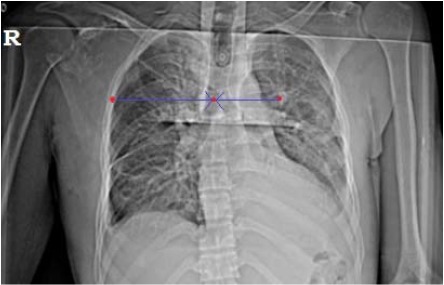


**Fig. 2: CT with 3D reconstruction images showing scapulothoracic dissociation with comminuted fracture of scapula and acromioclavicular joint disruption and associated avulsion fractures of the spinous processes of vertebrae (D3-D5):**
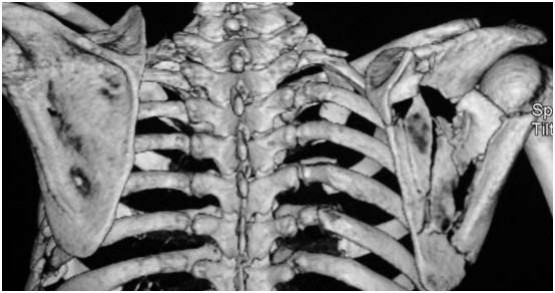


**Fig. 3: CT scan axial image revealing fracture of multiple spinous processes with the fractured fragments drawn towards the side of the scapulothoracic dissociation demonstrating the avulsion fracture and direction of force.**